# Preparation of Nanobubbles Carrying Androgen Receptor siRNA and Their Inhibitory Effects on Androgen-Independent Prostate Cancer when Combined with Ultrasonic Irradiation

**DOI:** 10.1371/journal.pone.0096586

**Published:** 2014-05-05

**Authors:** Luofu Wang, Miao Zhang, Kaibin Tan, Yanli Guo, Haipeng Tong, Xiaozhou Fan, Kejing Fang, Rui Li

**Affiliations:** 1 Department of Urology, Daping Hospital & Institute of Surgery Research, Third Military Medical University, Chongqing, China; 2 Department of Ultrasound, Southwest Hospital, Third Military Medical University, Chongqing, China; 3 Department of Ultrasound, Xinqiao Hospital, Third Military Medical University, Chongqing, China; Innsbruck Medical University, Austria

## Abstract

**Objective:**

The objective of this study was to investigate nanobubbles carrying androgen receptor (AR) siRNA and their *in vitro* and *in vivo* anti-tumor effects, when combined with ultrasonic irradiation, on androgen-independent prostate cancer (AIPC).

**Materials and Methods:**

Nanobubbles carrying AR siRNA were prepared using poly-L-lysine and electrostatic adsorption methods. Using C4-2 cell activity as a testing index, the optimal irradiation parameters (including the nanobubble number/cell number ratio, mechanical index [MI], and irradiation time) were determined and used for transfection of three human prostate cancer cell lines (C4-2, LNCaP, and PC-3 cells). The AR expression levels were investigated with RT-PCR and Western blot analysis. Additionally, the effects of the nanobubbles and control microbubbles named SonoVue were assessed via imaging in a C4-2 xenograft model. Finally, the growth and AR expression of seven groups of tumor tissues were assessed using the C4-2 xenograft mouse model.

**Results:**

The nanobubbles had an average diameter of 609.5±15.6 nm and could effectively bind to AR siRNA. Under the optimized conditions of a nanobubble number/cell number ratio of 100∶1, an MI of 1.2, and an irradiation time of 2 min, the highest transfection rates in C4-2, LNCaP, and PC-3 cells were 67.4%, 74.0%, and 63.96%, respectively. In the C4-2 and LNCaP cells, treatment with these binding nanobubbles plus ultrasonic irradiation significantly inhibited cell growth and resulted in the suppression of AR mRNA and protein expression. Additionally, contrast-enhanced ultrasound showed that the nanobubbles achieved stronger signals than the SonoVue control in the central hypovascular area of the tumors. Finally, the anti-tumor effect of these nanobubbles plus ultrasonic irradiation was most significant in the xenograft tumor model compared with the other groups.

**Conclusion:**

Nanobubbles carrying AR siRNA could be potentially used as gene vectors in combination with ultrasonic irradiation for the treatment of AIPC.

## Introduction

It is well established that prostate cancer is dependent on androgens [1]. Therefore, androgen deprivation has been the main treatment for advanced prostate cancer. With this therapy, however, the disease rapidly progresses to androgen-independent prostate cancer (AIPC) in most patients [2], [3]. Currently, there are no effective long-term therapies for AIPC. Developing new treatment strategies for AIPC remains attractive but is a challenging problem in clinical oncology. Previous studies have shown that the growth of AIPC is dependent on the androgen receptor (AR). Thus, blocking AR expression has great potential for the treatment of AIPC [4].

Studies have demonstrated that suppressing AR expression with RNA interference (RNAi) technology is an effective way to inhibit the growth of prostate cancer cells, indicating that this method may have the potential to overcome hurdles associated with AIPC treatment [5]. In our previous studies, AR double-stranded RNA (dsRNA) with a high specificity for AR genes was designed to block the expression of AR in AIPC cells and to inhibit cell growth [6]. However, this type of gene therapy approach is difficult to apply in a clinical setting due to low gene transfection efficiency. One of the key reasons for low transfection efficiency is the lack of an effective, noninvasive *in vivo* targeted gene delivery system. In recent years, ultrasound-destructible microbubbles have been shown to be a promising method for gene therapy. Indeed, ultrasound-mediated microbubble destruction can not only improve gene transfection efficiency but can also be used for the tissue-specific delivery of therapeutic agents [7], [8].

With the development of ultrasound molecular imaging and nanotechnology, the size of microbubbles continues to decline, and micro- to nano-scale sizes are now achievable. Nanobubbles offer several advantages in targeted gene transfection [9]. For example, nanobubbles are characterized by strong penetrating power and stable performance, which allow them to enter tumor tissues through the tumor vasculature [10]. Therefore, in this study, we combined RNAi technology with nanotechnology by preparing nanobubbles carrying AR small interfering RNA (siRNA). The physical properties of these bubbles were then assayed. Furthermore, the prepared nanobubbles were used for the siRNA transfection of AIPC cells together with ultrasonic irradiation treatment to induce the release of the siRNA transcripts. The *in vitro* transfection efficiency was systematically evaluated. Additionally, the anti-tumor efficacy of the nanobubbles was evaluated using imaging studies and a tumor growth inhibition assay in a mouse xenograft prostate cancer model. The results presented here provide experimental support for the use of nanobubbles carrying AR siRNA in combination with ultrasonic irradiation as a potential effective therapy for AIPC.

## Materials and Methods

### Synthesis of AR siRNA

Based on the previously determined 19-bp target sequence of AR cDNA, AR siRNA was synthesized with the Silencer siRNA Construction Kit (Ambion, Austen City, USA) [6]. Cy3-labeled AR siRNA was then synthesized using the Silencer siRNA Cy3 Labeling Kit (Ambion) for fluorescence tracing studies. The concentration of the synthesized siRNA was determined using a spectrophotometer, and the siRNAs were diluted to 50 µM for use in this study.

### Preparation of nanobubbles carrying AR siRNA and characterization of their physical properties

A suspension of lipid excipients, including dipalmitoylphosphatidylcholine (DPPC), dipalmitoylphosphatidylethanolamine (DPPE), dipalmitoylphosphatidic acid (DPPA), and dipalmitoylphosphatidylglycerol (DPPG) (in a molar ratio of 90∶2∶5∶3), was prepared in polyethylene glycol (in a molar ratio of 94∶6). The suspension was aliquoted into 1-ml vials and lyophilized. The lyophilized suspensions were rehydrated with 1 ml of hydration solution with 50% glucose, propylene glycol and glycerin by volume in 8∶1∶1. Perfluoropropane (C_3_F_8_) was then added to the vials to replace the air, and an ST-B series amalgamator (AT&M Biomaterials Co. Ltd., Beijing, China) was used to prepare the blank nanobubbles with an operating frequency greater than 4,500 rpm and an oscillation time of 90 s [11].

A poly-L-lysine (PLL) solution (1 mg/ml) was prepared with sterile double-distilled water. The PLL solution was then mixed with the blank nanobubbles at a 1∶1 (v/v) ratio and incubated at 4°C for 30 min. The mixture was then washed twice with phosphate-buffered saline (PBS) to remove any unbound PLL. Cy3-labeled AR siRNA was added to this nanobubble solution at a 1∶1 (v/v) ratio, and the mixture was incubated at 4°C for 30 min. Then, 200 µl of PBS was added to the nanobubble mixture, and the suspension was washed by centrifugation twice at 600 rpm for 3 min to remove any unbound AR siRNA, after which nanobubbles labeled with both AR siRNA and PLL were obtained. The nanobubble solution and the aqueous solution containing the unbound siRNA were then boiled for 5 min, after which the optical density was measured at 260 nm using a spectrophotometer. The number of nanobubbles was measured using a hemocytometer [10]. The following formula was used to calculate the siRNA loading capability:
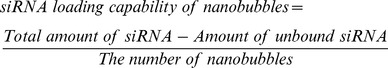



The size, distribution, and zeta potential of both the blank and prepared nanobubbles with AR siRNA were measured with a Zetasizer Nano ZS90 particle size analyzer (Malvern Instruments Ltd, Worcestershire, UK). The shape and dispersion of these nanobubbles were also examined using fluorescence microscopy (OLYMPUS LX71, Olympus Corporation, Tokyo, Japan).

### Cell lines and culture conditions

Three human prostate cancer cell lines were used in the transfection studies: LNCaP (androgen-dependent cells with AR expression, ATCC, Manassas, VA, USA), C4-2 (AIPC cells with AR expression, ViroMed Laboratories Inc., Minnetonka, MN, USA), and PC-3 (without AR expression, ATCC, Manassas, VA, USA). All the cell lines were cultured in RPMI-1640 medium containing 10% fetal bovine serum in a 5% CO_2_ incubator at 37°C. Routine passages were performed with trypsin digestion. One day before treatment with ultrasonic irradiation, all the cells were seeded in culture plates at a confluency of 50%.

### Optimization of irradiation conditions for transfection of nanobubbles carrying AR siRNA

To avoid nonspecific negative effects on cell activity, the appropriate concentration range for the blank nanobubbles and nondestructive irradiation conditions were determined prior to the *in vitro* cell experiments.

Ultrasonic irradiation was performed with the PHILIPS iU22 ultrasound system (Royal Dutch Philips Electronics Ltd, The Netherlands). The ultrasound probe was fixed with a bracket; the radiating surface faced vertically upward and was covered evenly with ultrasonic coupling gel; a 24-well plate with 1.5×10^5^ C4-2 cells cultured in each well was placed horizontally on the radiating surface of the probe to ensure that each well was completely exposed to the radiating source; the nanobubble suspension was added to each well and adequately mixed; and ultrasonic irradiation was performed with variations in several parameters, including the concentration of blank nanobubbles (assessed as the nanobubble number/cell number ratio), the mechanical index (MI, a reflection of the ultrasound energy calculated by dividing the peak negative acoustic pressure by the square root of the frequency), and the irradiation time. In detail, to evaluate the impact of the blank nanobubble concentration, six groups were established based on their nanobubble number/cell number ratio, which was 0, 45∶1, 90∶1, 135∶1, 180∶1, or the control group (normal cells without any treatment as control); the transducer frequency was 1.0–5.0 MHz, the duration was 30 s, and the MI was 1.0. An additional five groups were established with varying MIs, namely, 0.1, 0.6, 1, 1.4, or the control group (normal cells without any treatment as control); the transducer frequency was 1.0–5.0 MHz, the duration was 30 s, and the nanobubble number/cell number ratio was 100∶1. Five groups were established to evaluate the impact of the duration, which was 10 s, 30 s, 1 min, 2 min, or the control group (normal cells without any treatment as control); the transducer frequency was 1.0–5.0 MHz, the MI was 1.0, and the nanobubble number/cell number ratio was 100∶1. After ultrasonic irradiation, the cells were cultured for 24 h, and their cell growth was assayed using a blood counting instrument; the cell counts were used as a testing index to evaluate the effects of these parameters on cell activity. All the assays were repeated at least three times, and a range of nondestructive conditions for *in vitro* ultrasonic irradiation was determined.

### Transfection efficiency assay in prostate cancer cells

LNCaP, C4-2, and PC-3 cells were seeded in 24-well plates at a density of 5×10^4^ cells per well and cultured overnight. Nanobubbles carrying Cy3-labeled AR siRNA were then added to the wells of each cell culture plate and transfected into these three cell lines under the predetermined range of nondestructive conditions. The transfection efficiency was then assessed using fluorescence microscopy. The percentage of fluorescent cells (transfection rate) was determined by flow cytometry. Briefly, cells were resuspended after trypsin digestion to generate a single-cell suspension. Flow cytometry (FACSCalibur, Becton Dickinson, NJ, USA) was used to measure the fluorescence intensity of 10,000 cells, and the results were analyzed using FlowJo 7.65 software. Cells were gated to calculate the percentage of transfected cells within the total cell population, enabling determination of the optimized parameters for achieving the highest transfection efficiency. All the analyses were repeated at least three times.

### Cell growth inhibition assay

Each of the three prostate cancer cell lines was divided into six cell treatment groups according to the reagents and conditions used (Table 1). All the groups were transfected using the aforementioned ultrasonic irradiation method under the appropriate parameters: frequency of 1.0–5.0 MHz, mechanical index (MI) of 1.2, and duration of exposure to B-mode ultrasound of 2 min. The Cell Counting Kit-8 (CCK-8) was used daily from the first to the sixth day after transfection to assess cell growth. Each group had 10 equivalent testing wells and 2 negative control wells. The absorbance values at 450 nm [D (450 nm)] were assayed with a microplate reader (Model 550, Bio-Rad, USA). A cell growth curve was obtained by graphing time (X-axis) versus the respective absorbance value (Y-axis), and the cell growth inhibition rate (IR) was calculated using the following formula:
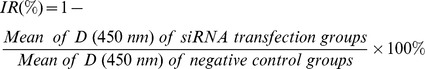



**Table 1:** Groups of experiments and corresponding treatment.

Groups	Treatment	Abbreviation
1	Bare AR siRNA + blank nanobubbles	siRNA+NB
2	Nanobubbles carrying AR siRNA	siRNA-NB
3	Bare AR siRNA + ultrasonic irradiation	siRNA+US
4	Blank nanobubbles + ultrasonic irradiation	NB+US
5	Bare AR siRNA + blank nanobubbles + ultrasonic irradiation	siRNA+NB+US
6	Nanobubbles carrying AR siRNA + ultrasonic irradiation	siRNA-NB+US
7*	Nanobubbles carrying nonsense siRNA + ultrasonic irradiation	NSsiRNA-NB+US

* Group 7 was not designed for *in vitro* experiment.

In addition, the cell morphology 48 h after transfection was observed using fluorescence microscopy.

### Detection of AR mRNA expression level by RT-PCR

LNCaP and C4-2 cells were transfected using the aforementioned ultrasonic irradiation method with the above parameters. Total RNA was extracted from all the groups 48 h after ultrasonic irradiation. An RT-PCR analysis was conducted using an RNA PCR Kit (AMV Ver. 3.0, Kyoto, Japan), and glyceraldehyde phosphate dehydrogenase (GAPDH) was used as a control. The primer sequences (5′→3′) for AR were AAG CCA TTG AGC CAG GTG TAG TG (upstream) and AAC CAG ATG AGG GGC GAA GTA GA (downstream), and the primer sequences for GAPDH were ACC CAT CAC CAT CTT CCA GGA G (upstream) and GAA GGG GCG GAG ATG ATG AC (downstream). These primers amplified a 275-bp AR fragment and a 159-bp GAPDH fragment, respectively. The reverse transcription conditions were 50°C for 30 min, 99°C for 5 min, and 5°C for 5 min. The PCR conditions were 94°C for 2 min; 32 cycles of 94°C for 30 s, 57°C for 30 s, and 72°C for 1 min; and 72°C for 10 min. The RT-PCR products were loaded on a 2% agarose gel. After electrophoresis, the bands were visualized using a Bio-Rad GelDoc 2000 gel imaging system (Bio-Rad, CA, USA) and were densitometrically analyzed using ImageJ software (NIH, http://rsb.info.nih.gov/ij/). All the analyses were repeated five times.

### Detection of AR protein expression level by Western blot analysis

LNCaP and C4-2 cells were transfected using the aforementioned ultrasonic irradiation method with the optimized parameters. Total cellular protein was extracted from all the groups using a cell lysis buffer 48 h after the ultrasonic irradiation. The protein concentration was determined using the Bradford assay. A 50-µg aliquot of each cell lysate was separated on a 12% sodium dodecyl sulfate-polyacrylamide gel electrophoresis (SDS-PAGE) gel and transferred to a polyvinylidene fluoride (PVDF) membrane. The membranes were blocked with 5% nonfat dry milk for 4 h at room temperature and incubated with a primary antibody (mouse monoclonal antibody against human AR; Santa Cruz Biotechnology, California, USA) at a 1∶400 dilution at 37°C for 4 h. The membranes were then washed with Tris-buffered saline/Tween (TBST) buffer and incubated with a secondary antibody (goat anti-mouse IgG; Zsjqbio, Beijing, China) (1∶1200 dilution) at 37°C for 2 h. An enhanced chemiluminescence (ECL) reagent was then used to detect the protein bands by exposing the blots to autoradiographic film. A quantitative image analysis was conducted using Quantity One 4.52 software (Bio-Rad). GAPDH was used to normalize the samples. All the analyses were repeated five times.

### Imaging of nanobubbles carrying AR siRNA in a mouse xenograft model

This animal study was approved by the Laboratory Animal Welfare and Ethics Committee of the Third Military Medical University, China. Five male SCID mice weighing 22–25 g (5–7 weeks old) were xenografted with 3×10^6^ C4-2 prostate cancer cells in 100 µl of Matrigel (BD Biosciences, Basel, Switzerland). Tumors were allowed to become established and grow to a diameter of 10–15 mm. The animals were then anesthetized with an intraperitoneal injection of 100 µl of 1% pentobarbital sodium solution and then secured in a prone position. Two-dimensional ultrasound images of the xenografted tumors were acquired using a PHILIPS iU22 ultrasound system. Nanobubbles carrying AR siRNA were then administered to the mice via tail vein injection at a dose of 5 ml/kg body weight. The parameters used to obtain the ultrasound images were as follows: probe frequency  = 5–12 MHz; MI = 0.4; and gain  = 70%. The probe was fixed, and contrast-enhanced dynamic images were captured using ultrasound workstation software. Micron-sized mircrobubbles named SonoVue contrast agent (Bracco, Milan, Italy) was used as a control under the same conditions. A quantitative analysis was performed using Philips QLAB 8.1 software (Royal Dutch Philips Electronics Ltd, The Netherlands).

### *In vivo* tumor growth inhibition assay

This animal study was approved by the Laboratory Animal Welfare and Ethics Committee of the Third Military Medical University, China. Fifty-six male SCID mice weighing 22–25 g (5–7 weeks old) were xenografted with 3×10^6^ C4-2 cells in 100 µl of Matrigel. The animal study was approved by the Animal Ethics Committee of China Pharmaceutical University. Tumors were allowed to become established and to grow to a diameter of 7–10 mm. The animals bearing tumor xenografts were then randomly divided into seven groups (eight animals per group). The treatments used for Groups 1–6 are listed in Table 1. Group 7 was administered nanobubbles carrying nonsense siRNA + ultrasonic irradiation. In detail, the doses of nanobubbles carrying AR siRNA (approximately 1.6×10^6^/µl), blank nanobubbles, and saline solution were 5 ml/kg body weight. The bare AR siRNA dosage was 100 µg per mouse. All the nanobubbles, bare AR siRNA, and saline solution were administered as an intravenous bolus via a tail vein. The mice in Groups 3–7 were then anesthetized by isoflurane and secured in a prone position. Immediately after injection, the tumor xenografts were treated using a Philips iU22 ultrasound system. The perfusion of nanobubbles into the tumor was visualized in real time using ultrasound (5–12 MHz) at low acoustic power (MI 0.4) to minimize nanobubble destruction. Upon visualization of the nanobubbles within a tumor, the nanobubbles were burst by increasing the MI to 1.2 with a frequency of 1–5 MHz. An ultrasound exposure duration of 30 min was applied to ensure that all the nanobubbles were destroyed. During the treatment, the transducer was applied in a circular motion to ensure that the entire tumor xenograft received ultrasound exposure. For all the animals, the treatment was performed three times, once every 3 d (on day 0, day 3, and day 6). The maximum diameter (a, mm) and the shortest diameter (b, mm) of each tumor were measured every 3 d beginning on the first treatment day (day 0) and ending 21 days after the first treatment (day 21). The tumor volume (TV) was calculated according to the following formula: TV = 1/2×a×b^2^. The relative tumor volume (RTV) was then calculated using the following formula: RTV = TV_n_/TV_0_×100%, where TV_n_ is the tumor volume measured on day n and TV_0_ is the tumor volume on day 0. The tumor growth curve was plotted based on the RTV. On day 21, all the animals were sacrificed, and the tumors were exercised and weighed. The tumor growth inhibition rate (TGIR) was calculated using the following formula: TGIR = (1−TWt/TWc)×100%, where TWt and TWc are the mean tumor weights in the treated and control groups, respectively.

### Detection of AR mRNA expression in prostate tumors by qRT-PCR

Total RNA was isolated from tumor tissues following the instructions supplied with the SuperScript VILO RT-PCR Synthesis Kit (Invitrogen). The concentration and integrity of the RNA were determined by spectrophotometric analysis at A260 and A280. Approximately 500 ng of total RNA was reverse-transcribed with Superscript III (Invitrogen) using N6 random primers. Approximately 2 µl of cDNA was amplified using one-step real-time PCR on an Applied Biosystems 7500 FAST machine with the standard program. The relative expression levels of AR mRNA were calculated using the formula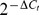, where.
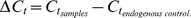


### Detection of AR protein expression level in prostate tumors by Western blot analysis

Xenograft tumor tissue was homogenized with 1 ml of radioimmunoprecipitation assay (RIPA) buffer containing protease inhibitor and centrifuged at 12,000 g for 10 min at 4°C, and the supernatant was stored at −80°C. Total protein was separated on a 10% SDS-PAGE gel and transferred to a PVDF membrane at 100 V for 50 min. The Western blot steps were the same as those described for the *in vitro* experiment.

### Statistical analysis

The statistical analysis was performed with SPSS 13.0 software (SPSS Inc., Chicago, IL, USA). Quantitative data are presented as the means ± standard deviation (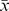 ± SD). A one-way analysis of variance (ANOVA) was used to compare the means of multiple groups (n>2). Independent-samples t-tests were used to compare the means of each treatment group and its relative control group. A P-value (P) <0.05 was considered statistically significant.

## Results

### Physical properties of nanobubbles carrying AR siRNA

**Figure 1:**
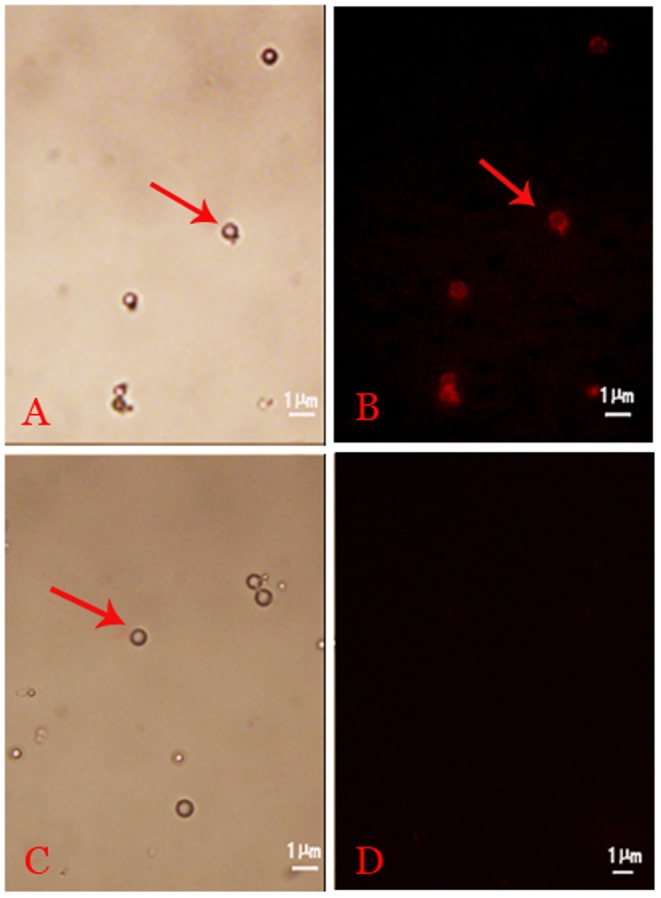
Nanobubbles with Cy3-labeled AR siRNA observed under fluorescence microscopy. (A) PLL nanobubbles under bright-field microscopy. (B) Obvious fluorescence was observed in the PLL nanobubbles. (C) Blank nanobubbles under bright-field microscopy. (D) No fluorescence was observed in the blank nanobubbles. The red arrows in the images indicate nanobubbles (1,000×).

**Figure 2:**
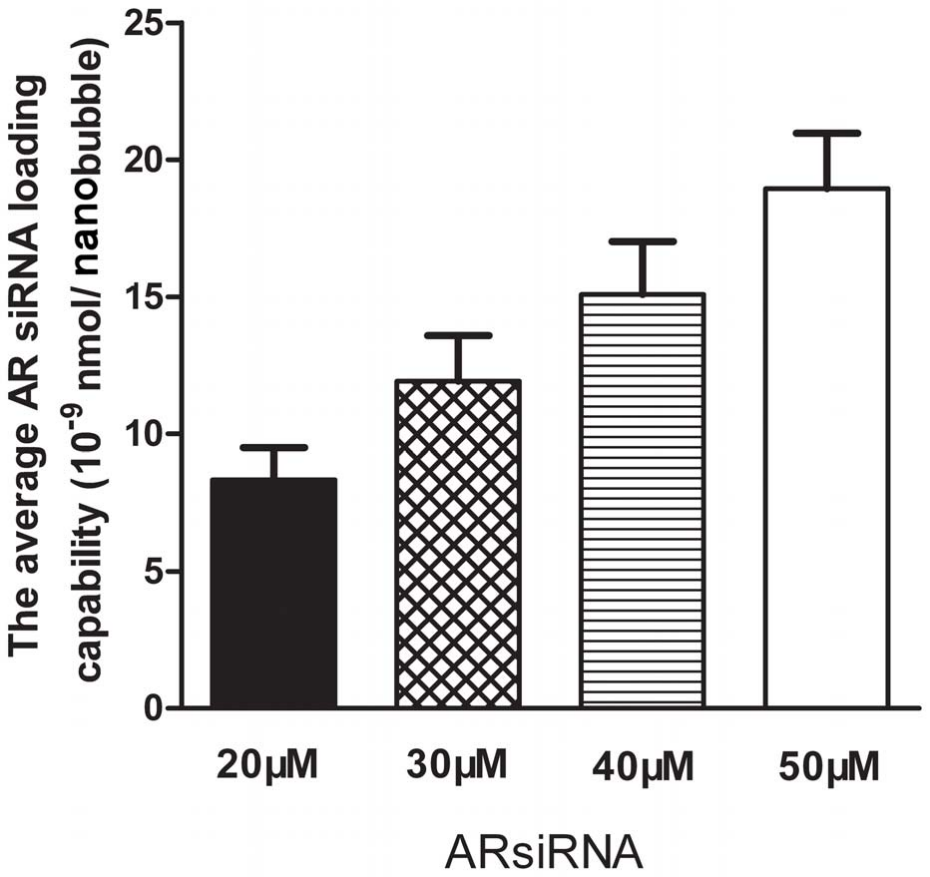
The average AR siRNA loading capacity of PLL nanobubbles varied in a dose-dependent manner.

Images obtained under bright-field microscopy showed that the nanobubbles were homogeneous in size and distributed evenly with no significant aggregation (Fig. 1A). The regular nanobubbles were observed under an optical microscope (1,000×) (Fig. 1C). Fluorescence microscopy confirmed that our siRNA labeling approach was effective; only nanobubbles conjugated with Cy3-labeled AR siRNA exhibited red fluorescence (Fig. 1B), and no fluorescence was observed with the regular nanobubbles (Fig. 1D). The results obtained with a particle size analyzer indicated that the nanobubbles had an average diameter of 609.5±15.6 nm (range, 269 to 1030 nm), and their zeta potential was −29.9±6.6 mV. The blank nanobubbles had an average diameter of 509.3±45.19 nm (range, 324 to 930 nm), and the zeta potential of these bubbles was −27.3±6.4 mV. As shown in Fig. 2, the amount of siRNA loaded increased in a dose-dependent manner. The average AR siRNA loading capability of the nanobubbles was (18.94±0.35)×10^−9^ nmol/nanobubble.

### Optimal irradiation conditions for transfection

**Figure 3:**
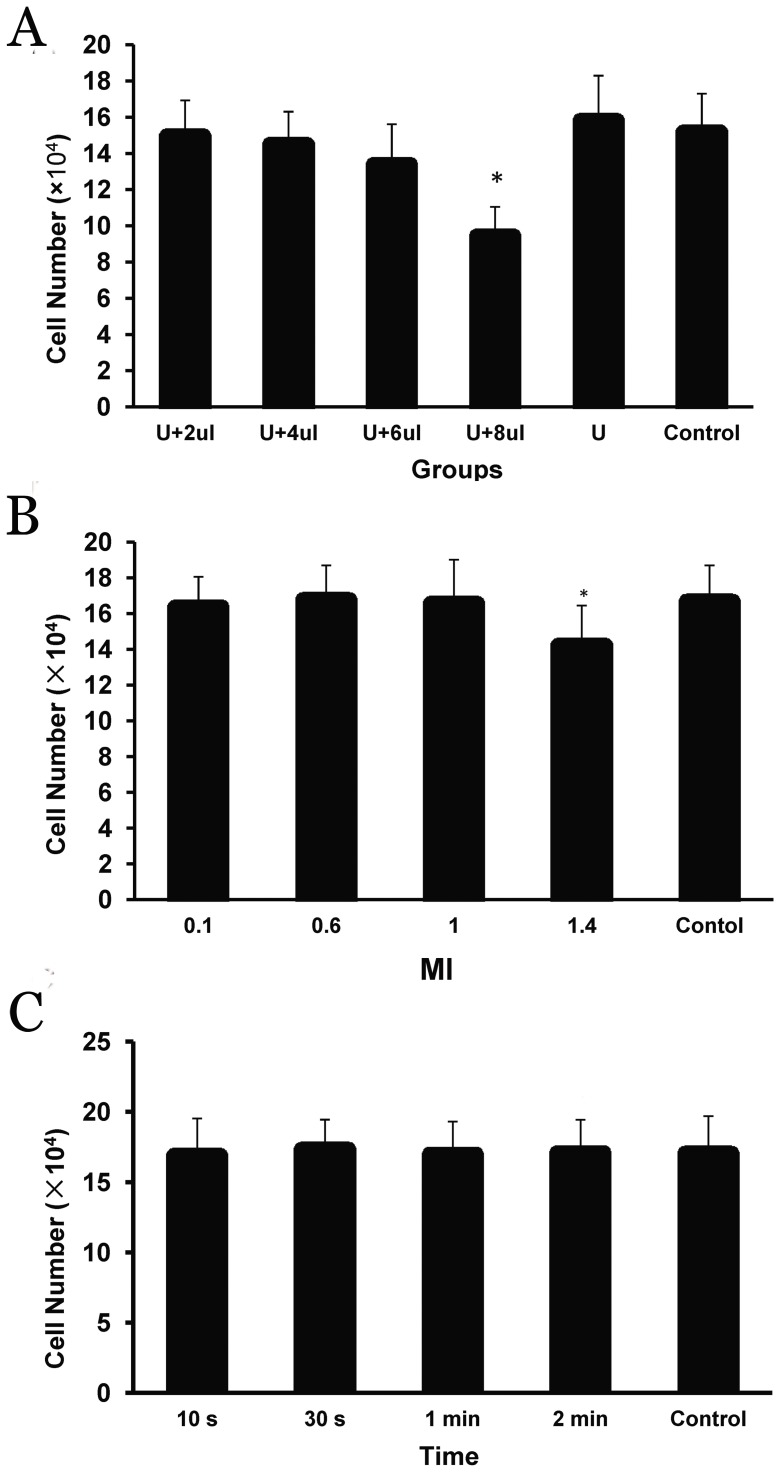
The impacts of various ultrasonic irradiation parameters on C4-2 cell growth. (A) The impact of the nanobubble concentration (with an MI of 1.0 and an irradiation time of 30 s), U: exposed to ultrasound; (B) the impact of MI, with a nanobubble number/cell number ratio of 100∶1 and an irradiation time of 30 s; and (C) the impact of irradiation time, with a nanobubble number/cell number ratio of 100∶1 and an MI of 1.0. Cells were counted 24 h after ultrasonic irradiation. All the assays were repeated three times. The data are presented as the means ± SD (n = 3). Asterisks (*) represent the level of significance compared with the control (P<0.05).

Fig. 3 shows the impacts of various parameters of ultrasonic irradiation on C4-2 cell growth. A volume of nanobubbles greater than 6 µl (the nanobubble number/cell number ratio was 135∶1) resulted in a significant decrease in cell growth (P<0.05 compared with the control) (Fig. 3A). An MI greater than 1.4 also caused a significant inhibition of cell growth (P<0.05 compared with the control) (Fig. 3B). Nevertheless, the impact of irradiation time on cell growth was found to be rather low; there were virtually no significant differences in cell growth between the groups treated with different irradiation times (Fig. 3C). Thus, the nondestructive irradiation conditions were ultimately determined to be a nanobubble number/cell number ratio<135∶1 (<6 µl of nanobubbles), an MI<1.4, and an irradiation time ≤2 min.

### Transfection efficiency assay in prostate cancer cells

**Figure 4:**
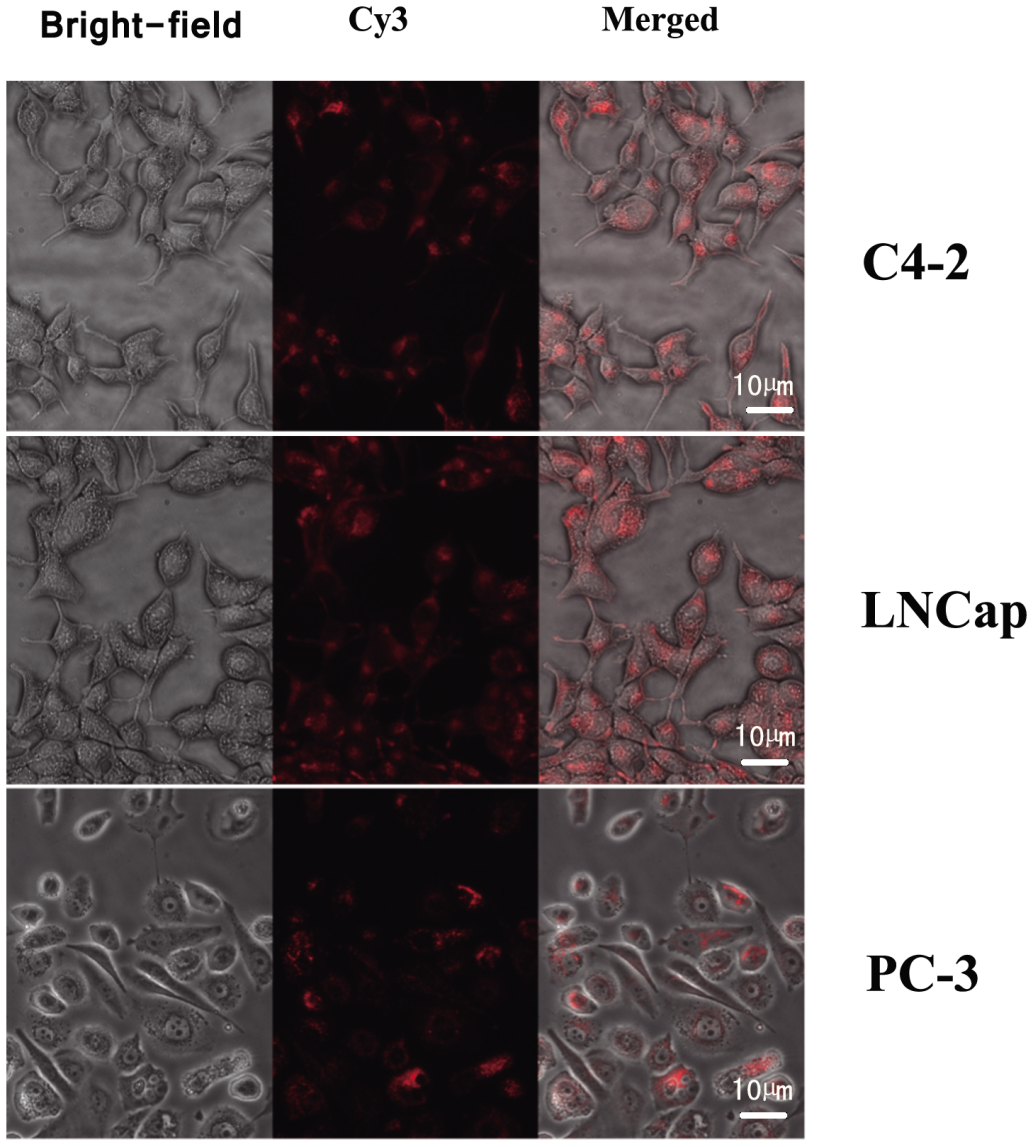
Images of three prostate cancer cell lines transfected with Cy-3-labeled AR siRNA observed using fluorescence microscopy. Red fluorescence in the cytoplasm indicates that the Cy3-labeled AR siRNA was successfully transfected (400× magnification).

Red fluorescence (indicative of Cy3-labeled AR siRNA) was observed in most parts of the cytoplasm in all three prostate cancer cell lines, but it was not detected in the nucleus (Fig. 4). These results indicate that the Cy3-labeled AR siRNA was successfully transfected. The percentages of transfected cells in these cell lines under different ultrasonic irradiation conditions are shown in Table 2.

**Table 2:** Transfection efficiency of three tumor cell lines subjected to ultrasonic irradiation under various conditions.

Irradiation parameters			Percentage of transfected cells (%)		
			C4-2	LNCaP	PC-3
Time = 30 s	MI	0.1	2.0±0.3*	4.0±0.4*	1.3±0.5*
		0.4	13.2±2.2*	11.3±1.8*	11.3±2.5*
		0.8	44.1±3.7*	47.9±3.9*	39.8±5.8*
		1.0	63.4±5.3*	68.6±6.1*	58.6±6.2*
		1.2	67.4±5.7*	74.0±5.9*	63.9±7.6*
		control	0.3±0.0	0.28±0.0	0.3±0.0
MI = 1.0	Time	10 s	32.1±2.7**	33.4±5.5**	29.3±4.3**
		30 s	42.2±5.2**	47.7±6.5**	40.5±6.1**
		1 min	59.2±3.7**	65.3±4.8**	55.3±4.5**
		2 min	61.0±5.3**	64.5±5.1**	56.1±5.1**
		control	0.28±0.0	0.3±0.0	0.3±0.0

The data are presented as the means ± SD (n = 3). * P<0.05 and ** P<0.01 indicate the level of significance compared with the control.

As shown in Table 2, when nanobubbles carrying AR siRNA were administered at a nanobubble number/cell number ratio of 100∶1 (5 µl of nanobubbles), the percentage of transfected cells increased along with increases in MI and irradiation time. The highest transfection efficiency was achieved with an MI of 1.2 and an irradiation time of 2 min. Therefore, the optimized parameters for achieving the highest transfection efficiency were as follows: 5 µl of nanobubbles, an MI of 1.2, and an irradiation time of 2 min; the following *in vitro* assays were performed using these parameters.

### Cell growth inhibition

**Figure 5:**
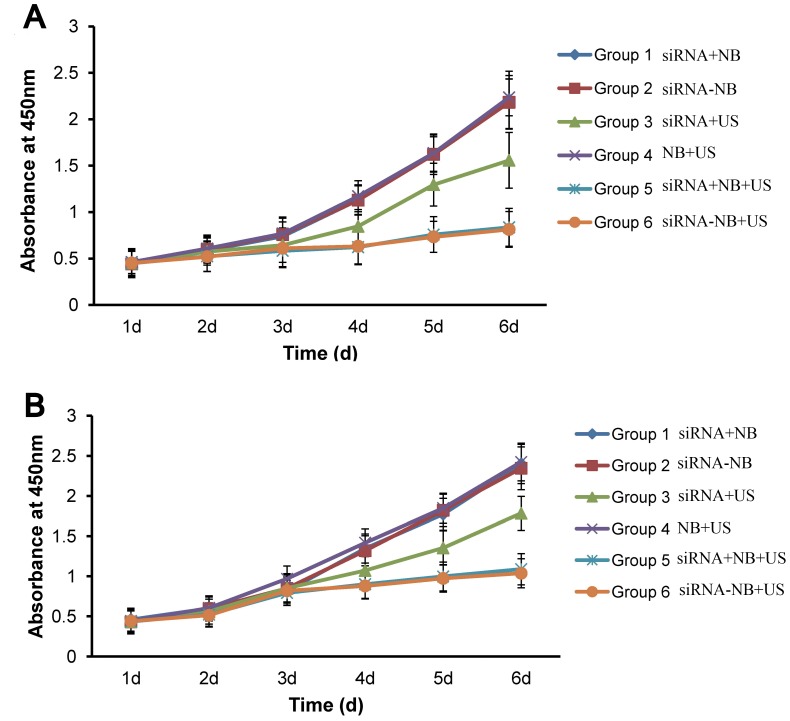
Cell growth curves of C4-2 cells (A) and LNCaP cells (B) after transfection. Significant inhibition of cell growth was observed in Groups 3, 5, and 6 for both cell lines. The data are presented as the means ± SD (n = 10). Groups 1–6 represent bare AR siRNA + blank nanobubbles, nanobubbles carrying AR siRNA, bare AR siRNA + ultrasonic irradiation, blank nanobubbles + ultrasonic irradiation, bare AR siRNA + blank nanobubbles + ultrasonic irradiation, and nanobubbles carrying AR siRNA + ultrasonic irradiation, respectively.

The CCK-8 analysis from the first to the sixth day after transfection indicated that cell growth was inhibited in Groups 3, 5, and 6 of the C4-2 and LNCaP cells (Fig. 5A and 5B). On the sixth day after transfection, the C4-2 cells in Group 6 displayed the highest growth inhibition rate at 64.7%. However, no significant cell growth inhibition was observed in the PC-3 cells, and the growth inhibition rate in Group 6 was just 0.9% (Table 3).

**Table 3:** Cell growth inhibition in three prostate cancer cell lines on the sixth day after transfection.

Groups	C4-2 (%)	LNCaP (%)	PC-3 (%)
1	1.2±0.1	1.0±0.1	0.9±0.1
2	2.4±0.3	3.2±0.2	1.0±0.2
3	31.3±1.7	23.1±0.9	1.0±0.1
4	0	0	0
5	63.6±3.6	55.1±2.2	0.9±0.1
6	64.7±2.8^*Δ^	57.2±2.4^*Δ^	0.9±0.1

The data are presented as the means ± SD (n = 10). An asterisk (*) indicates that the growth inhibition of C4-2 and LNCAP cells in Group 6 was significantly different from that in Groups 1, 2, and 4 (P<0.05). A triangle (Δ) indicates that the growth inhibition of C4-2 and LNCaP cells in Group 6 was significantly different from that of the PC-3 cells in the same group (P<0.05).

### AR mRNA expression levels assayed by RT-PCR

**Figure 6:**
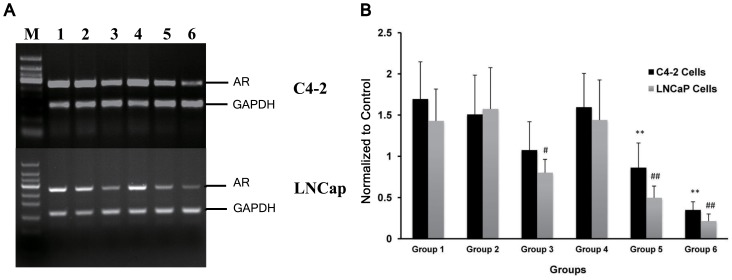
Electrophoresis results for RT-PCR products. Lanes 1–6 represent Groups 1–6, respectively (Group1: siRNA+NB, Group2: siRNA-NB, Group3: siRNA+US, Group4: NB+US, Group5: siRNA+NB+US, Group6: siRNA-NB+US). The sizes of the markers (the M lane, from bottom to top) are 50, 100, 150, 200, 250, 300, 350, 400, and 450 bp. GAPDH served as an internal reference. The highest suppression of AR mRNA expression was observed in Group 6. (A) and (B): Bands of RT-PCR products and the results of image quantification; the data in the histograms represent the means ± SD of five independent experiments. (* P<0.05 and ** P<0.01 vs. Group 1 C4-2 cells; # P<0.05 and ## P<0.01 vs. Group 1 LNCaP cells).

The agarose gel electrophoresis results following RT-PCR are shown in Fig. 6A. Bands of 275 bp (AR) and 159 bp (GAPDH) were observed in all groups of both C4-2 and LNCaP cells. AR bands were not observed in any of the PC-3 groups because PC-3 cells do not express AR. The results of the quantitative analysis (Fig. 6B) showed that in both cell lines, AR mRNA expression was significantly suppressed in Groups 3, 5, and 6 compared with the control (Group 1). The highest suppression was achieved in Group 6.

### Post-transfection AR protein expression levels assayed by Western blot analysis

**Figure 7:**
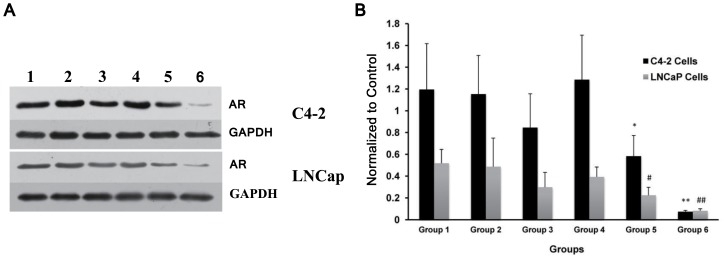
Western blot results of AR protein expression in C4-2 and LNCaP cells. Lanes 1–6 represent Groups 1–6, respectively (Group1: siRNA+NB, Group2: siRNA-NB, Group3: siRNA+US, Group4: NB+US, Group5: siRNA+NB+US, Group6: siRNA-NB+US). GAPDH protein served as an internal reference. The highest suppression was observed in Group 6. (A) and (B): Immuno-bands and the results of image quantification; the data in the histograms represent the means ± SD of five independent experiments. (* P<0.05 and ** P<0.01 vs. Group 1 C4-2 cells; # P<0.05 and ## P<0.01 vs. Group 1 LNCaP cells).

The Western blot results are shown in Fig. 7. The AR protein expression was suppressed in Groups 3, 5, and 6 of the C4-2 and LNCaP cells. The highest suppression was observed in Group 6, which was consistent with the RT-PCR results.

### Imaging of nanobubbles carrying AR siRNA in the C4-2 prostate cancer xenograft model

**Figure 8:**
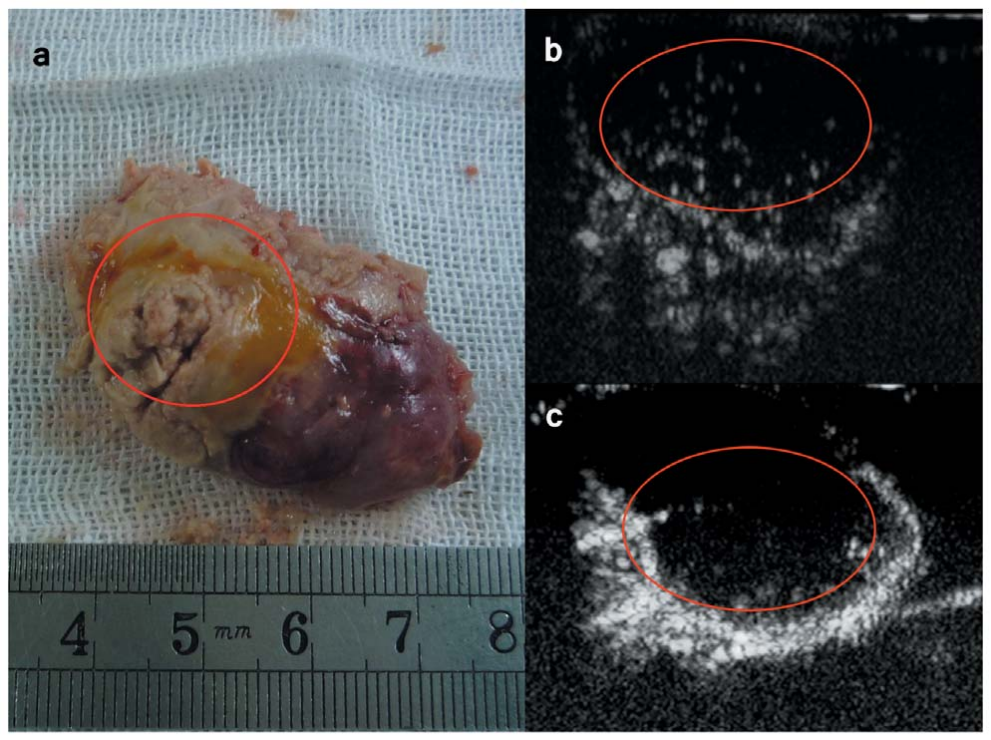
Contrast-enhanced dynamic images of nanobubbles carrying AR siRNA in a C4-2 prostate cancer xenograft model. (A) A xenograft tumor specimen; (B) ultrasonic imaging of a C4-2 xenograft after the injection of nanobubbles carrying AR siRNA using a Philips iU22 ultrasound system; and (C) ultrasonic imaging of a C4-2 xenograft after the injection of SonoVue micron-sized microbubbles as a control. The region marked with a red circle in the three panels represents the central hypovascular area of the tumor. The nanobubbles carrying AR siRNA achieved stronger signals than SonoVue in this area.

Contrast-enhanced dynamic images of nanobubbles carrying AR siRNA are shown in Fig. 8. The results indicated that the nanobubbles achieved stronger signals than SonoVue in the central hypovascular area of the tumors. The imaging data analysis (Table 4) demonstrated that these nanobubbles had significant increases in peak intensity and imaging duration compared with the SonoVue control (P<0.05). In the surrounding tumor area, the nanobubbles also displayed significantly longer imaging durations than SonoVue (P<0.05).

**Table 4:** Imaging data of nanoscale microbubbles carrying AR siRNA in a C4-2 prostate cancer xenograft model.

	Agents	Imaging Time (s)	Peak Time (s)	Peak Intensity (dB)	Duration (min)
Surrounding tumor area	Nanobubbles carrying AR siRNA	3.0±0.9	8.1±1.2	16.1±4.2	7.8±1.4*
	SonoVue	3.2±0.7	7.8±1.0	17.2±3.6	4.2±1.1
Central tumor area	Nanobubbles carrying AR siRNA	7.2±1.9	13.1±1.5	6.5±0.4*	5.6±1. 2*
	SonoVue	6.8±0.9	12.6±1.1	3.6±0.2	2.1±0.4

The data are presented as the means ± SD (n = 5).

^*^P<0.05 indicates the level of significance compared with the SonoVue control.

### *In vivo* tumor growth inhibition assay

**Figure 9:**
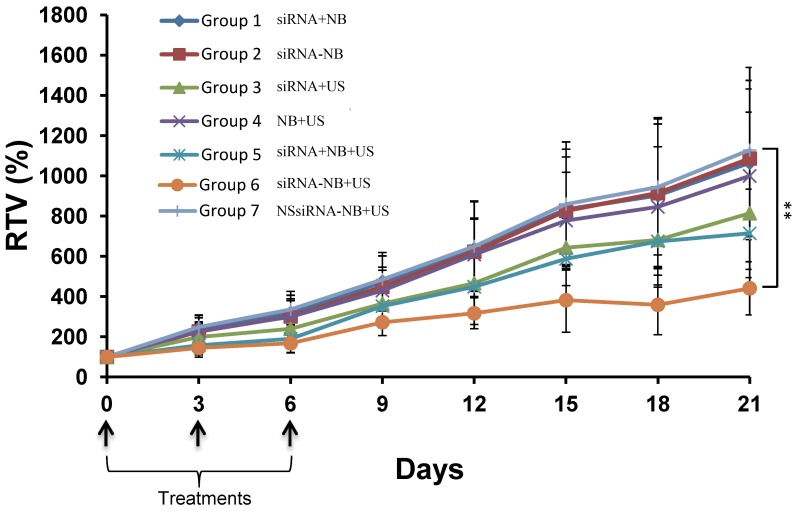
The tumor growth curves for Groups 1–7. The data are presented as the means ± SD (n = 8). For all animals, treatments were performed three times, once every 3 d (on day 0, day 3, and day 6, as indicated by arrows). Significant inhibition of tumor growth was observed in Group 6. Asterisks (**) represent the level of significance compared with the control (Group 7) (P<0.01).

The tumor growth curves for Groups 1–7 are shown in Fig. 9. The tumor growth in Group 6 was significantly inhibited compared with that of the control (P<0.01). Nevertheless, there were no significant differences between Groups 3 and 5 and the control (P>0.05), although tumor growth inhibition was also observed in Groups 3 and 5. The TGIRs of Groups 1–7 were as follows: 5.51%, 3.73%, 27.82%, 11.39%, 36.70%, 60.88%, and 0%, respectively. During the experiment, no deaths occurred in the control or treated animals.

### AR mRNA expression levels assessed by qRT-PCR in prostate tumor xenografts

**Figure 10:**
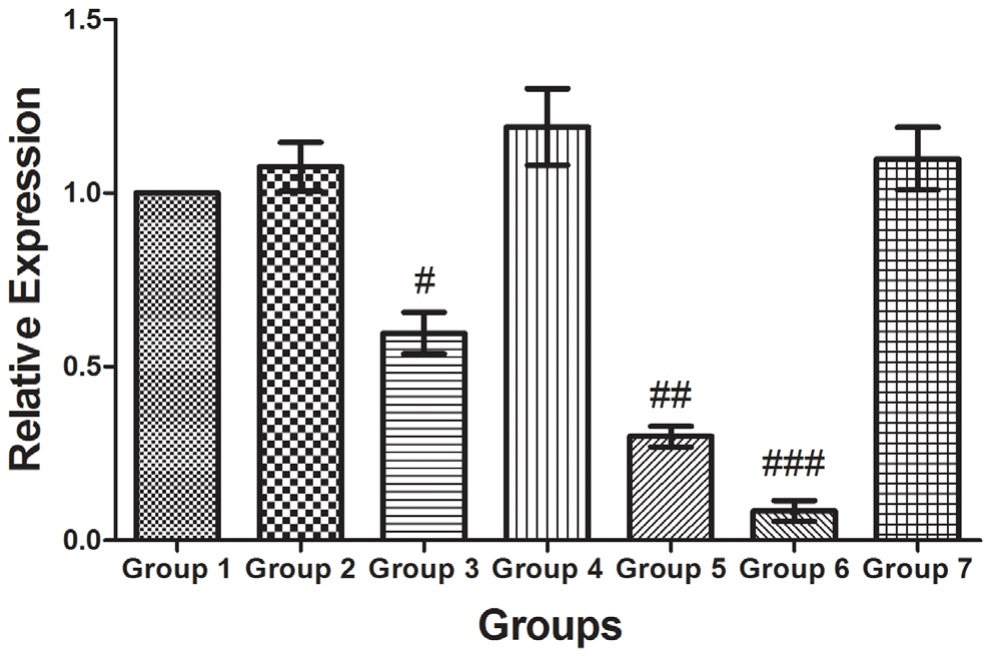
qRT-PCR quantification of AR expression in the animals in Groups 1–7. The lowest relative expression of AR mRNA was observed in Group 6 (siRNA-NB+US). The data in the histograms represent the means ± SEM of 8 animals (each sample was assayed in triplicate). # P<0.05, ## P<0.01, and ### P<0.001 compared with Group 1 (independent-samples t-test).

The results of qRT-PCR showed that the relative expression levels of AR mRNA were 59.67±6%, 29.89±4%, and 8.46±2% in Groups 3, 5, and 6, respectively, compared with Group 1. The lowest relative expression was found in Group 6 (Fig. 10).

### AR protein expression level in prostate tumors assayed by Western blot analysis

**Figure 11:**
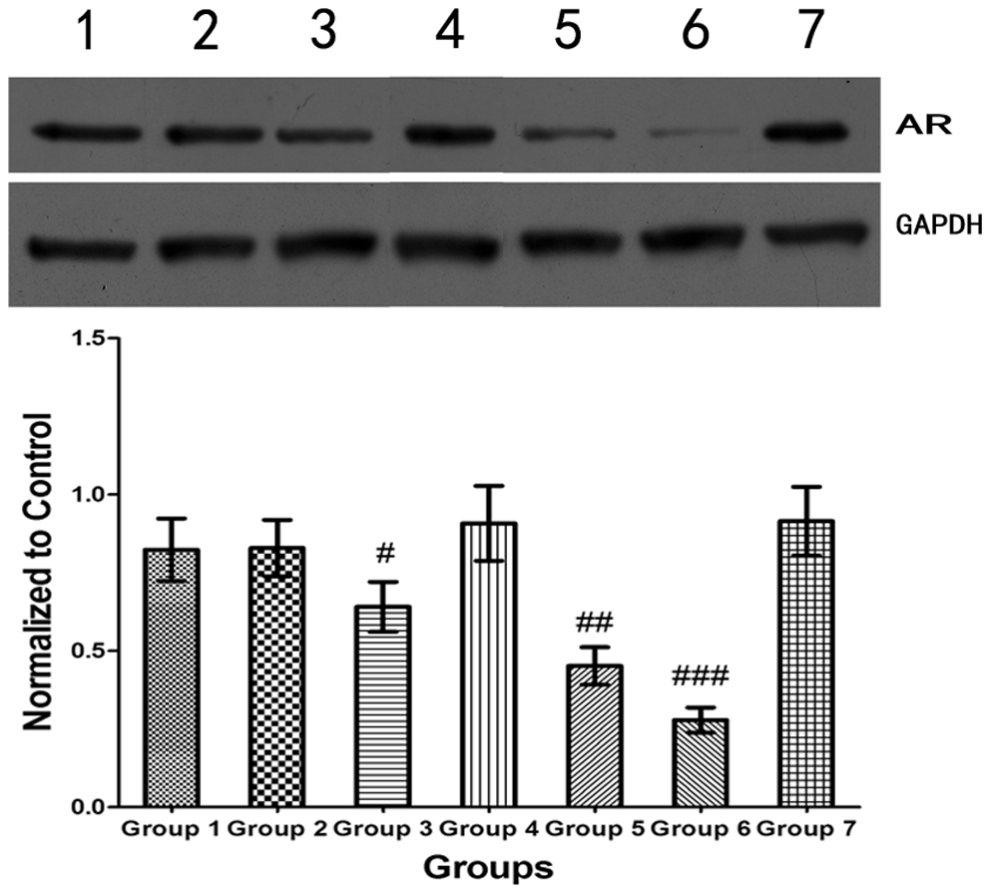
Western blot quantification of AR expression in the animals in Groups 1–7. GAPDH protein served as the internal reference. The highest suppression was observed in Group 6 (siRNA-NB+US). The data in the histograms represent the means ± SEM of 8 animals (each sample was assayed in triplicate). # P<0.05, ## P<0.01, and ### P<0.001 compared with Group 1 (independent-samples t-test).

The Western blot results are shown in Fig. 11. The AR protein expression was significantly suppressed in Groups 3, 5, and 6 compared with Group 1. The highest suppression was observed in Group 6, which was consistent with the qRT-PCR results.

## Discussion

To date, various gene therapy treatments utilizing viral vectors have been proposed. However, the use of viral vectors is a major concern because this vector system is associated with numerous safety concerns, including toxicity, immunogenicity, and tumorigenicity. Thus, the clinical application of viral vectors has been greatly restricted. The construction of an efficient, safe, and controllable *in vivo* gene delivery system has been a highly debated and challenging endeavor in the gene therapy community [12]. The present study shows that ultrasound-mediated microbubble destruction may have the potential to become a new approach for targeted gene transfection. Other studies have also shown that ultrasound-destructible microbubbles not only enhanced gene transfection efficiency but were also potentially useful as a novel *in vivo* gene transfection vector for the delivery of any antisense oligonucleotide or DNA fragment [13], [14]. Thus, ultrasound-destructible microbubbles show great promise for clinical applications in the future [15], [16].

Despite this promise, some challenges remain in the application of conventional ultrasound-destructible microbubbles for gene or drug delivery to treat cancer. Most notably, the diameter of conventional ultrasound-destructible microbubbles, which ranges from 1–10 µm, likely prevents the bubbles from passing through the tumor vascular wall into the tumor tissues because the maximum pore size of the tumor vascular wall is 380–780 nm [17]. This size discrepancy can affect the release and transfection of carried genes, ultimately decreasing the anti-tumor efficacy of these micro-scaled, ultrasound-destructible microbubbles [18], [19]. To address this size problem, researchers have proposed that nanobubbles may be a better alternative to conventional microbubbles as a gene or drug carrier for the treatment of tumors [9]. Nanobubbles have a size of less than 1000 nm. Compared with conventional microscale microbubbles, nanobubbles exhibit enhanced tissue penetration, a feature that allows them to access the inside of tumor tissues and achieve real target imaging and treatment [15]. Based on the results of this study, the ultrasound-destructible nanobubbles carrying AR siRNA that we prepared could not only enhance the imaging effect of transplanted tumor, but also distribute more widely in the tumor, compared with the control microbubbles - SonoVue. In other normal tissues and organs, such as the liver and the kidney, nanobubbles had longer imaging duration than microbubbles while there was no obvious different distribution between them [20]. Additionally, recent studies have shown that nanobubbles under ultrasonic irradiation can also enhance gene transfection efficiency [9], [15]. Therefore, in theory, nanobubbles show great potential for gene therapy.

The difficulty of developing nanobubbles with a high carrying capacity for highly stable drugs and genes prevents the application of ultrasound-destructible nanobubbles as a drug or gene transfection vector. Currently, electrostatic binding and biotin/avidin binding are the two main methods of combining genes with microbubbles [21], [22]. Electrostatic binding is not suitable for wide use due to its instability and low encapsulation rate. Biotin/avidin binding, although stable, cannot be used for nanobubbles because of the large molecular weight of biotin/avidin, which can result in a significant increase in bubble size. Some researchers have utilized the PLL method, which can be regarded as an improved method of electrostatic binding [23]. PLL contains a polycation chain with a positively charged surface, and the prepared nanobubbles have a negatively charged surface. After PLL binding via static electricity, the surface of the nanobubble has a positive charge and can combine with the negatively charged siRNA via electrostatic adsorption. In this study, nanobubbles carrying AR siRNA were prepared based on the above mechanism. Nanobubbles with an average size of 609.5 nm exhibited stable binding to siRNA, as indicated by the finding that the loading capability of the carried nucleic acids remained high after repeated washes with PBS. These results suggest that PLL is an effective method for preparing nanobubbles carrying nucleic acids.

Currently, however, the application of nanobubbles carrying genes to inhibit tumor growth remains in the exploratory stage. Many key issues, such as the preparation method, stability, penetration capability, *in vivo* imaging properties, and transfection efficiency of nanobubbles, still need to be systematically investigated [19]. Therefore, when accompanied by ultrasonic irradiation, these bubbles facilitated a high transfection efficiency in AIPC cells, providing evidence that ultrasound-destructible nanobubbles may represent a viable option for gene therapy delivery to tumors *in vivo*.

Many studies have also shown that the intensity and duration of ultrasonic irradiation and the concentration of microbubbles can inhibit cell growth and even cause cell death [13]. This phenomenon may interfere with evaluations of the inhibitory effects of nanobubbles carrying siRNA on the growth of prostate cancer cells. Therefore, it is necessary to identify a nondestructive irradiation paradigm and an ideal concentration range of blank nanobubbles that do not interfere with cellular activity. Previous studies have demonstrated that the ratio of microbubble number to cell number is a suitable parameter for determining the ideal concentration of microbubbles. In addition, with a given concentration of microbubbles, the ultrasonic irradiation conditions are the main factor affecting the efficiency of gene transfection [24]. In this study, we evaluated the impacts of different MIs, irradiation times, and blank nanobubble concentrations on cell growth activities and ultimately optimized the irradiation conditions for transfection.

In cell growth inhibition assays, a significant inhibitory effect was observed in the groups of C4-2 and LNCaP cells treated with ultrasonic irradiation (Groups 3, 5, and 6), indicating that ultrasonic irradiation could effectively promote siRNA transfection. This finding is consistent with that of Haag P et al., who found that ultrasonic irradiation alone could effectively promote the silencing effects of oligodeoxynucleotides (ODNs) targeting AR receptors, consequently inhibiting the growth of prostate cancer cells [25]. This effect can likely be attributed to the ability of ultrasonic irradiation to increase the permeability of cell membranes, which could help ODNs penetrate cells and exert their inhibitory effects on cell growth. The achievement of the highest inhibition efficacy in Group 6 of the C4-2 and LNCaP cells demonstrated that ultrasonic irradiation with nanobubbles promoted efficient AR siRNA entry into cells, consequently suppressing the expression of AR mRNA and protein and ultimately inhibiting cell growth by silencing the AR receptor. These results were similar to those obtained with micro-scaled bubbles. The associated mechanism is likely based on the production of acoustic cavitation by nanoscale bubbles with ultrasonic irradiation, an effect that could increase the permeability of cell membranes and allow AR siRNA to enter cells for enhanced transfection and therapeutic efficacy [26]. In addition, local ultrasonic irradiation successfully destroyed nanobubbles and facilitated the targeted release of AR siRNA, enabling AR siRNA to accumulate locally in tumor tissues at a higher concentration and effectively exert a silencing function, which was initially demonstrated by the results of imaging studies and tumor growth inhibition assays in C4-2 xenograft models. The achievement of the highest suppression of AR mRNA and protein in Group 6 demonstrated that the ultrasound irradiation and the nanobubbles carrying AR siRNA functioned synergistically to inhibit the growth of AIPC tumors. Therefore, the nanobubbles that we produced may possess potential for the treatment of prostate cancer. However, this possibility requires further validation with future studies, and the safety and *in vivo* distribution of these nanobubbles will be key issues for investigation in subsequent studies. Thermal effect might exist in our experiment, but its effect was trivial. In Group 4, tumors were treated with ultrasonic irradiation and we did not observe obvious inhibition of the tumors. Thermal effect is remarkable in high intensity focused ultrasound (HIFU) and tissue thermal damage is approximately linearly dependent on exposure time. HIFU treatment often needs to maintain a certain amount of time, which usually lasts for 120–240 minutes [27]. We used low intensity ultrasound and irradiation time last only 30 minutes, so if thermal effect existed, it had almost no effect on tumor growth.

In summary, the AR siRNA-coupled nanobubbles that we prepared in this study possessed ideal *in vitro* and *in vivo* properties, including nano-scale size, good stability, and strong *in vivo* penetration ability into xenografted tumors. *In vitro* studies confirmed that, coupled with ultrasonic irradiation, the nanobubbles carrying AR siRNA could significantly inhibit the growth of C4-2 cells and LNCaP cells. The results of the *in vivo* tumor growth inhibition assay, qRT-PCR, and Western blot analysis also initially demonstrated the inhibitory efficacy of the nanobubbles carrying AR siRNA against C4-2 xenograft tumors. Thus, this study not only identified an effective gene carrier but also provides a solid foundation for further investigations into the feasibility of nanobubbles with ultrasonic irradiation as a therapeutic approach for the treatment of refractory prostate cancers, such as AIPC.
